# Circulating oxylipin and bile acid profiles of dexmedetomidine, propofol, sevoflurane, and S-ketamine: a randomised controlled trial using tandem mass spectrometry

**DOI:** 10.1016/j.bjao.2022.100114

**Published:** 2022-12-12

**Authors:** Aleksi Nummela, Lauri Laaksonen, Annalotta Scheinin, Kaike Kaisti, Tero Vahlberg, Mikko Neuvonen, Katja Valli, Antti Revonsuo, Markus Perola, Mikko Niemi, Harry Scheinin, Timo Laitio

**Affiliations:** 1 Turku PET Centre, University of Turku and Turku University Hospital, Turku, Finland; 2 Department of Internal Medicine, Turku University Hospital, Turku, Finland; 3 Department of Peri-operative Services, University of Turku and Turku University Hospital, Turku, Finland; 4 Department of Clinical Medicine, Biostatistics, Intensive Care and Pain Medicine, University of Turku and Turku University Hospital, Turku, Finland; 5 Department of Clinical Pharmacology, University of Helsinki, Helsinki, Finland; 6 Individualized Drug Therapy Research Program, Faculty of Medicine, University of Helsinki, Helsinki, Finland; 7 Department of Psychology and Speech-Language Pathology, and Turku Brain and Mind Center, University of Turku, Turku, Finland; 8 Department of Cognitive Neuroscience and Philosophy, School of Bioscience, University of Skövde, Skövde, Sweden; 9 Department of Clinical Pharmacology, HUS Diagnostic Center, Helsinki University Hospital, Helsinki, Finland; 10 Research Program for Clinical and Molecular Metabolism, Faculty of Medicine, University of Helsinki, Helsinki, Finland; 11 Integrative Physiology and Pharmacology, Institute of Biomedicine, University of Turku, Turku, Finland; 12 Finnish Institute for Health and Welfare, Helsinki, Finland

**Keywords:** bile acids, dexmedetomidine, lipidomics, oxylipins, propofol, sevoflurane, S-ketamine

## Abstract

**Background:**

This exploratory study aimed to investigate whether dexmedetomidine, propofol, sevoflurane, and S-ketamine affect oxylipins and bile acids, which are functionally diverse molecules with possible connections to cellular bioenergetics, immune modulation, and organ protection.

**Methods:**

In this randomised, open-label, controlled, parallel group, Phase IV clinical drug trial, healthy male subjects (*n*=160) received equipotent doses (EC_50_ for verbal command) of dexmedetomidine (1.5 ng ml^−1^; *n*=40), propofol (1.7 μg ml^−1^; *n*=40), sevoflurane (0.9% end-tidal; *n*=40), S-ketamine (0.75 μg ml^−1^; *n*=20), or placebo (*n*=20). Blood samples for tandem mass spectrometry were obtained at baseline, after study drug administration at 60 and 130 min from baseline; 40 metabolites were analysed.

**Results:**

Statistically significant changes *vs* placebo were observed in 62.5%, 12.5%, 5.0%, and 2.5% of analytes in dexmedetomidine, propofol, sevoflurane, and S-ketamine groups, respectively. Data are presented as standard deviation score, 95% confidence interval, and *P*-value. Dexmedetomidine induced wide-ranging decreases in oxylipins and bile acids. Amongst others, 9,10-dihydroxyoctadecenoic acid (DiHOME) –1.19 (–1.6; –0.78), *P*<0.001 and 12,13-DiHOME –1.22 (–1.66; –0.77), *P*<0.001 were affected. Propofol elevated 9,10-DiHOME 2.29 (1.62; 2.96), *P*<0.001 and 12,13-DiHOME 2.13 (1.42; 2.84), *P*<0.001. Analytes were mostly unaffected by S-ketamine. Sevoflurane decreased tauroursodeoxycholic acid (TUDCA) –2.7 (–3.84; –1.55), *P*=0.015.

**Conclusions:**

Dexmedetomidine-induced oxylipin alterations may be connected to pathways associated with organ protection. In contrast to dexmedetomidine, propofol emulsion elevated DiHOMEs, oxylipins associated with acute respiratory distress syndrome, and mitochondrial dysfunction in high concentrations. Further research is needed to establish the behaviour of DIHOMEs during prolonged propofol/dexmedetomidine infusions and to verify the sevoflurane-induced reduction in TUDCA, a suggested neuroprotective agent.

**Clinical trial registration:**

NCT02624401.

As functionally diverse molecules, measuring oxylipin and bile acids may deepen our understanding of the possible immunomodulatory, bioenergetic, and organ-protective properties of dexmedetomidine, propofol, sevoflurane, and S-ketamine.^1^, ^2^, ^3^ Previously, we discovered that a 1 h exposure to these agents induced unique changes in the metabolic profiles of healthy subjects in the absence of perioperative confounding factors.^4^ Lipoprotein measurements and fatty acid ratios were altered in response to propofol and, to a lesser degree, dexmedetomidine. Dexmedetomidine affected glucose and ketone metabolism likely mirroring α_2_-adrenoceptor agonism. In response to S-ketamine, glucose and lactate increased and branched chain amino acids (isoleucine, leucine, and valine) decreased. In the sevoflurane group, analytes were relatively unaffected.

Oxygenated unsaturated fatty acids, oxylipins, are central poly-unsaturated fatty acid (PUFA) effectors in humans. Briefly, oxylipins possess immunomodulatory, anti- and pro-inflammatory, and vasoactive attributes and can affect cellular bioenergetics. The actions of specific oxylipins have been reviewed previously.^1^, ^2^, ^3^ Oxylipins are formed mainly from linoleic (LA), alpha-linoleic (α-LA), arachidonic (AA), and eicosapentaenoic (EPA) acids *via* three pathways: cyclooxygenase, lipoxygenase (LOX), and cytochrome P450 (CYP). Previously, we observed propofol-induced reductions in oxylipin precursors PUFA and LA relative to total fatty acids.^4^ Whether downstream oxylipin synthesis is also affected seems an interesting possibility.

Bile acids are cholesterol-derived nutritional detergents synthesised by the liver. Discoveries on the widespread distribution of bile acid precursors and receptors have sparked interest in their role as signalling molecules. Interestingly, neuroprotective effects of tauroursodeoxycholic acid (TUDCA) have been described.^5^ In neonatal animal models, learning and memory deficits were induced by repeated sevoflurane exposure, but the observed increase in hippocampal markers of endoplasmic reticulum stress and decrease in synaptic plasticity-associated proteins were reversed by administration of TUDCA.^6^

Whether and how oxylipins and bile acids are affected by anaesthetic agents and sedatives is largely unknown. In this explorative study, we aimed to investigate whether dexmedetomidine, propofol, sevoflurane, and S-ketamine acutely alter circulating oxylipin and bile acid profiles in healthy subjects.

## Methods

### Trial design and participants

This randomised, open-label, controlled, parallel group, Phase IV clinical drug trial (ClinicalTrials.gov identifier NCT02624401) was conducted at Turku PET Centre, University of Turku, Turku, Finland as a part of ‘The Neural Mechanisms of Anesthesia and Human Consciousness’ project (from January 2016 to March 2017), as predefined in the trial protocol. This study was approved by the Ethics Committee of the Hospital District of Southwest Finland and the Finnish Medicines Agency Fimea (EudraCT 2015-004982-10). This article adheres to the applicable Consolidated Standards of Reporting Trials (CONSORT) guidelines. A detailed description of the study methods and the CONSORT flow diagram have been published earlier.^4^, ^7^

A total of 160 healthy, ASA physical status Class 1 male subjects were randomly allocated to receive one of the following study treatments: dexmedetomidine (Dexdor 100 μg ml^−1^; Orion Pharma, Espoo, Finland; *n*=40), propofol (Propolipid 10 mg ml^−1^; Fresenius Kabi, Uppsala, Sweden; *n*=40), sevoflurane (Sevoflurane 100%; AbbVie, Espoo, Finland; *n*=40), S-ketamine (Ketanest-S 25 mg ml^−1^; Pfizer, Helsinki, Finland; *n*=20), or saline placebo (*n*=20). The inclusion criteria have been described earlier.^7^ In accordance with the Declaration of Helsinki, a written informed consent was obtained from all study subjects.

### Study drug administration and monitoring

The duration of study drug administration was 60 min. Subject preparation, monitoring, and the details of administration, including pharmacokinetic parameters, have been described earlier.^7^ Briefly, target-controlled infusion with a Harvard 22 syringe pump (Harvard Apparatus, South Natick, MA, USA) and STANPUMP software (www.opentci.org/code/stanpump) was used for dexmedetomidine, propofol, and S-ketamine administration.^4^ A Primus anaesthesia workstation (Drägerwerk AG & Co. KGaA, Lübeck, Germany) was used for sevoflurane administration and monitoring.

The targeted effective concentration at which 50% subjects were unresponsive to verbal command (EC_50_) was based on previous studies, as reported earlier: 1.5 ng ml^−1^ for dexmedetomidine, 1.7 μg ml^−1^ for propofol, 0.75 μg ml^−1^ for S-ketamine, and end-tidal target of 0.9% for sevoflurane.^8^, ^9^, ^10^ The data on monitored concentrations of dexmedetomidine, propofol, and sevoflurane and end-tidal concentration of sevoflurane have been published earlier.^7^

### Blood sampling

Arterial blood samples were collected at three time points: the first sample at baseline before study drug administration (Time point 1), the second at the end of 60 min study drug administration (Time point 2), and the third approximately 70 min after the cessation of study drug administration (Time point 3). Sample preparation, storage, and transfer were carried out, as described earlier.^4^ Immediately after sampling, the blood samples were cooled and protected from light. Cold centrifugation (+4°C) was used for plasma separation within 30 min of sampling, followed by sample division into amber tubes (Matrix™ 1.0mL 2D Screw Tubes Amper PP; Thermo Scientific, MA, USA). Amber tube samples were immediately frozen at –20°C and transferred to –70°C storage within the same day.

### Lipidomics analysis

Forty-six oxylipin and bile acid analytes were quantified by means of tandem mass spectrometry. A detailed description of the methodology can be found in Supplementary Appendix A.

### *In vitro* analysis

Because of the accompanying lipid emulsion in propofol formulation (Propolipid 10 mg ml^−1^), a dilution series of propofol emulsion was prepared in human plasma in ratios of 1:10, 1:100, 1:200, and 1:1000 (v/v). A blank human plasma served as control. Oxylipin levels in these samples were analysed using the same method as for the study samples.

### Statistical analysis

A formal power analysis was not considered applicable because of the exploratory nature of the study. Balanced permuted blocks were used for randomisation, as described previously.^7^

Of the 45 quantified analytes, 40 oxylipin and bile acid analytes were included in statistical analysis (24 and 16, respectively). Five metabolites (taurolithocholic acid, prostaglandin E2, prostaglandin D2 [PGD2], 5,6-dihydroxyeicosatetraenoic acid [DiHETE], and 8,9-dihydroxyeicosatrienoic acid [DHET]) were omitted from analysis because of the extensive number of values under the quantification limit (94.5%, 76.6%, 90.1%, 90.5%, and 92.6% of all values, respectively).

Logarithmic transformation was performed for metabolites with skewness >1 (100% of all metabolites). All metabolites were scaled to baseline standard deviation (sd). Zero values, including values under the detection limit, were omitted from the analysis (6.8% of all values included in the analysis). The statistical analysis was performed using repeated measures analysis of variance (anova) with each metabolite marker as outcome and time as a within factor and group as a between factor.^11^ Because all metabolites were analysed using separate anova models, there is no assumption concerning the dependency between metabolites. The mean differences in sd changes (95% confidence interval [CI]) between groups for all metabolites were estimated from a repeated measures model using group-by-time interaction effect. The drug–placebo and drug–drug group differences in sd changes were estimated between time points 1 *vs* 2 and 1 *vs* 3. The mean group difference in sd change units is referred to as the sd score (SDS). SDS was chosen instead of *z*-score to allow easy comparison to prior studies. To account for multiple testing (40 metabolites, 10 pairwise group comparisons, and two time-point comparisons), the *P*-values were Bonferroni corrected by a factor of 800, and an alpha threshold of 0.05 was used. Data are expressed as SDS (95% CI) between time points 1 *vs* 2, if not otherwise stated. Statistical analyses were carried out with SAS software (version 9.4; SAS Institute Inc., Cary, NC, USA).

For data visualisation, forest plots and line graphs were created using R (version 1.1.383; R Foundation for Statistical Computing, Vienna, Austria; https://www.R-project.org/) ggplot2 function (version 3.2.1; https://ggplot2.tidyverse.org).

**Fig 1:**
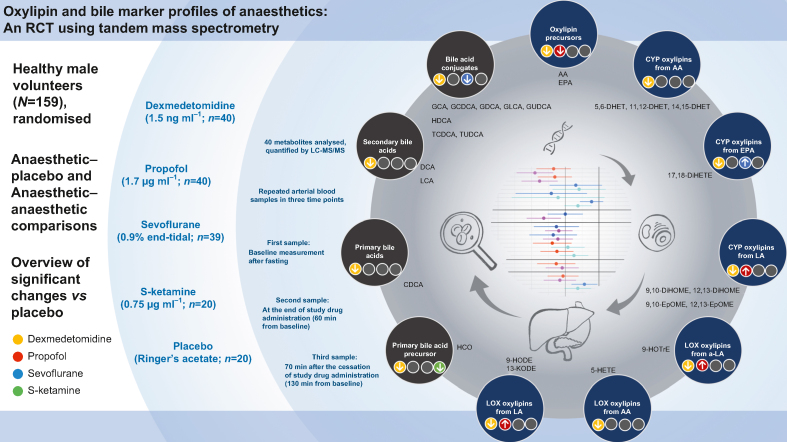
Infographic summarising the study methods and results. Modified from Nummela and colleagues.^4^ AA, arachidonic acid; CDCA, chenodeoxycholic acid; CYP, cytochrome P450 epoxygenase; DCA, deoxycholic acid; 5,6-DHET, 5,6-dihydroxy-8,11,14-eicosatrienoic acid; 11,12-DHET, 11,12-dihydroxy-5,8,14-eicosatrienoic acid; 14,15-DHET, 14,15-dihydroxy-5,8,11-eicosatrienoic acid; 17,18-DiHETE, 17,18-dihydroxy-5,8,11,14-eicosatetraenoic acid; 9,10-DiHOME, 9,10-dihydroxy-12-octadecenoic acid; 12,13-DiHOME, 12,13-dihydroxyoctadec-9-enoic acid; EPA, eicosapentaenoic acid; 9,10-EpOME, 9,10-epoxy-12-octadecenoic acid; 12,13-EpOME, 12,13-epoxy-9-octadecenoic acid; GCA, glycocholic acid; GCDCA, glycochenodeoxycholic acid; GDCA, glycodeoxycholic acid; GLCA, glycolithocholic acid; GUDCA, glycoursodeoxycholic acid; HCO, 7α-hydroxy-4-cholesten-3-one; HDCA, hyodeoxycholic acid; 5-HETE, 5-hydroxy-6,8,11,14-eicosatetraenoic acid; 9-HODE, 9-hydroxy-10,12-octadecadienoic acid; 9-HOTrE, 9-hydroxy-10,12,15-octadecatrienoic acid; 13-KODE, 13-keto-9,11,-octadecadienoic acid; LA, linoleic acid; α-LA, alpha-linoleic acid; LCA, lithocholic acid; LOX, lipoxygenase; TCDCA, taurochenodeoxycholic acid; TUDCA, tauroursodeoxycholic acid.

Figure 1 is an infographic that summarises the study methods and results.

## Results

Forty oxylipin and bile acids were analysed. Data from 159 subjects were evaluable. Samples of one subject in the sevoflurane group and two individual time-point samples in the placebo group were lost. The CONSORT flow diagram; baseline characteristics of the subjects; and the monitored concentrations of dexmedetomidine, propofol, sevoflurane, and S-ketamine have been published earlier.^47^ The study was completed as planned, and no significant changes in the vital signs were observed.

**Fig 2:**
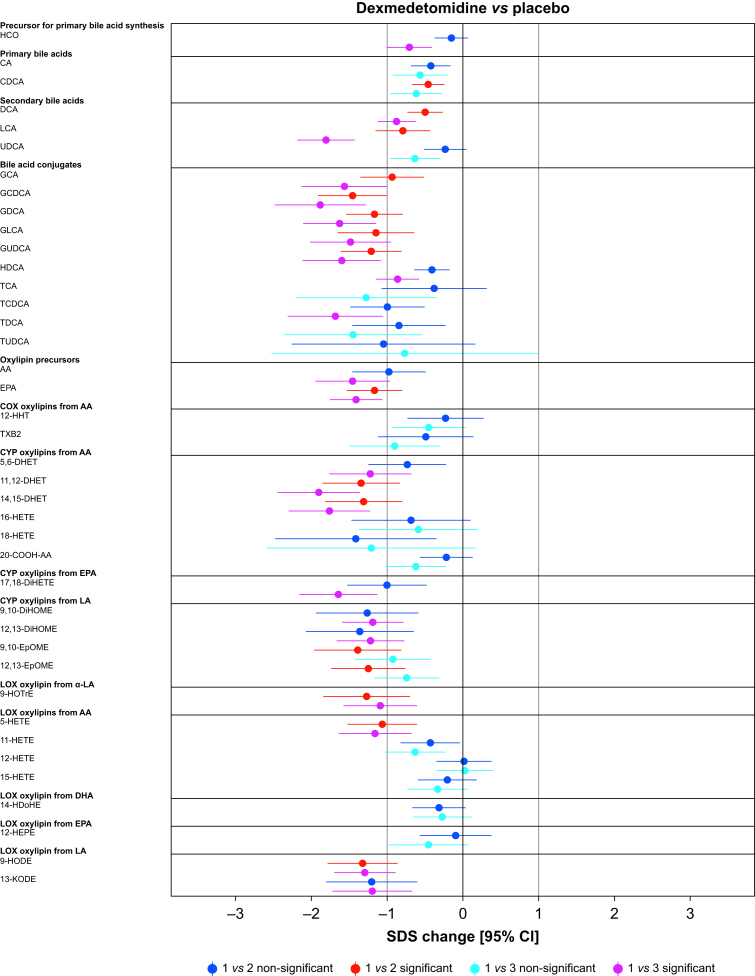

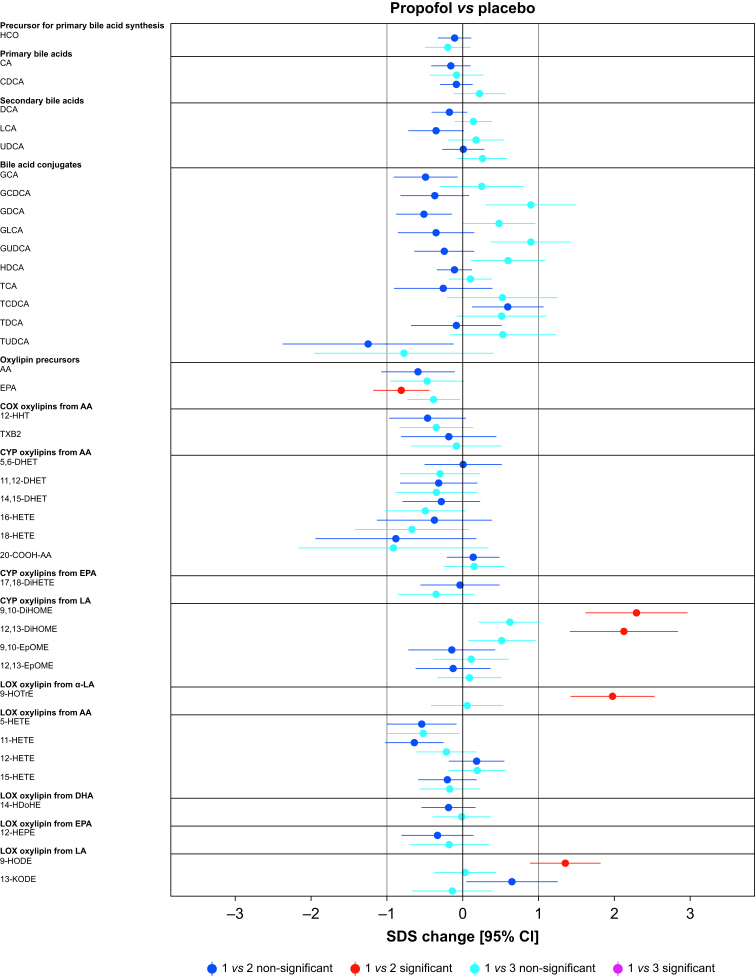
Forest plots: dexmedetomidine and propofol *vs* placebo, all analysed metabolites. Change is reported in SDS with 95% CIs. For all drug–drug comparisons, please refer to Supplementary Appendix B. The vertical lines depict 0 and 1 standard deviation thresholds. The colour coding represents the changes in time points 1 *vs* 2 and 1 *vs* 3; the significant changes are highlighted. AA, arachidonic acid; CA, cholic acid; CDCA, chenodeoxycholic acid; CI, confidence interval; 20-COOH-AA, 5,8,11,14-eicosatetraenedioic acid; DCA, deoxycholic acid; DHA, docosahexaenoic acid; 5,6-DHET, 5,6-dihydroxy-8,11,14-eicosatrienoic acid; 8,9-DHET, 8,9-dihydroxy-5,11,14-eicosatrienoic acid; 11,12-DHET, 11,12-dihydroxy-5,8,14-eicosatrienoic acid; 14,15-DHET, 14,15-dihydroxy-5,8,11-eicosatrienoic acid; 5,6-DiHETE, 5,6-dihydroxy-8,11,14,17-eicosatetraenoic acid; 17,18-DiHETE, 17,18-dihydroxy-5,8,11,14-eicosatetraenoic acid; 9,10-DiHOME, 9,10-dihydroxy-12-octadecenoic acid; 12,13-DiHOME, 12,13-dihydroxyoctadec-9-enoic acid; EPA, eicosapentaenoic acid; 9,10-EpOME, 9,10-epoxy-12-octadecenoic acid; 12,13-EpOME, 12,13-epoxy-9-octadecenoic acid; GCA, glycocholic acid; GCDCA, glycochenodeoxycholic acid; GDCA, glycodeoxycholic acid; GLCA, glycolithocholic acid; GUDCA, glycoursodeoxycholic acid; HCO, 7α-hydroxy-4-cholesten-3-one; HDCA, hyodeoxycholic acid; 14-HDoHE, 14-hydroxy docosahexaenoic acid; 12-HEPE, 12-hydroxy-5,8,10,14,17-eicosapentaenoic acid; 5-HETE, 5-hydroxy-6,8,11,14-eicosatetraenoic acid; 11-HETE, 11-hydroxy-5,8,12,14-eicosatetraenoic acid; 12-HETE, 12-hydroxy-5,8,10,14-eicosatetraenoic acid; 15-HETE, 15-hydroxy-5,8,11,13-eicosatetraenoic acid; 16-HETE, 16-hydroxy-5,8,11,14-eicosatetraenoic acid; 18-HETE, 18-hydroxy-5,8,11,14-eicosatetraenoic acid; 12-HHT, 12-hydroxy-5,8,10-heptadecatrienoic acid; 9-HODE, 9-hydroxy-10,12-octadecadienoic acid; 9-HOTrE, 9-hydroxy-10,12,15-octadecatrienoic acid; 9-HpODE, 9-hydroperoxyoctadeca-10,12-dienoic acid; 13-KODE, 13-keto-9,11,-octadecadienoic acid; LA, linoleic acid; α-LA, alpha-linoleic acid; LCA, lithocholic acid; PGD2, prostaglandin D2; PGE2, prostaglandin E2; SDS, sd score; TCA, taurocholic acid; TCDCA, taurochenodeoxycholic acid; TDCA, taurodeoxycholic acid; TUDCA, tauroursodeoxycholic acid; TXB2, thromboxane B2; UDCA, ursodesoxycholic acid.

**Fig 3:**
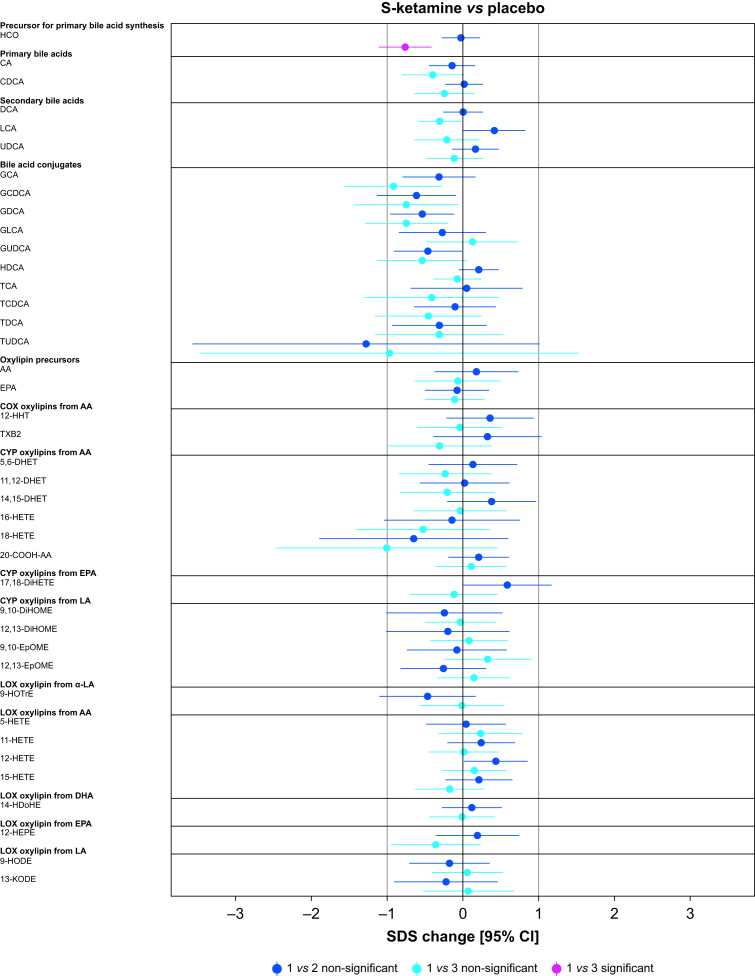

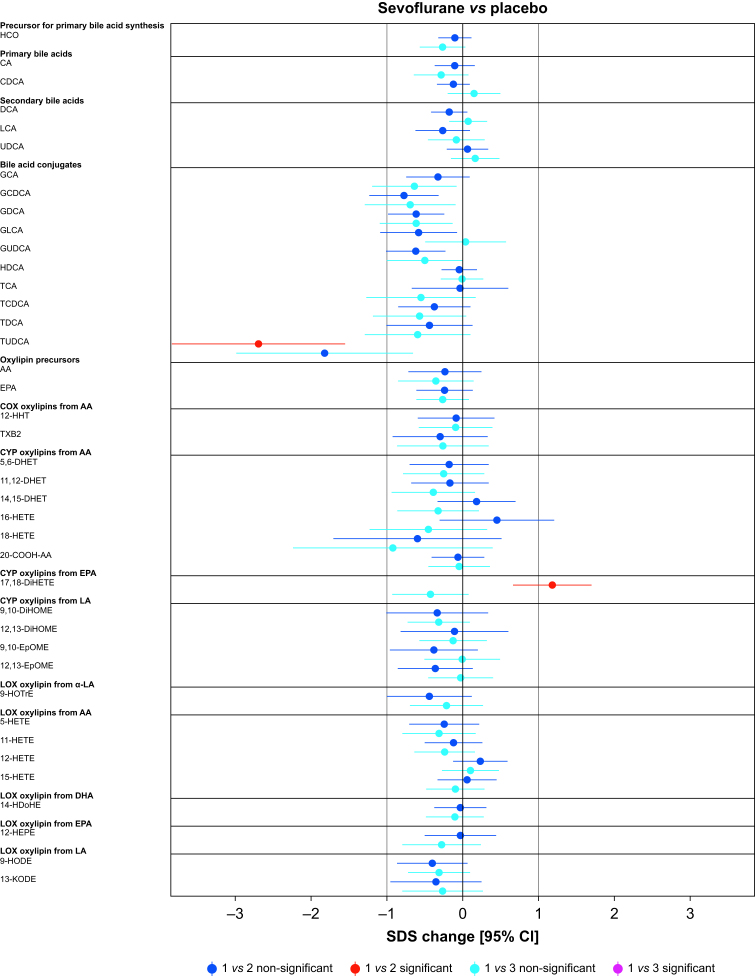
Forest plots: sevoflurane and S-ketamine *vs* placebo, all analysed metabolites. Change is reported in SDS with 95% CIs. For all drug–drug comparisons, please refer to Supplementary Appendix B. The vertical lines depict 0 and 1 standard deviation thresholds. The colour coding represents the changes in time points 1 *vs* 2 and 1 *vs* 3; the significant changes are highlighted. AA, arachidonic acid; CA, cholic acid; CDCA, chenodeoxycholic acid; CI, confidence interval; 20-COOH-AA, 5,8,11,14-eicosatetraenedioic acid; DCA, deoxycholic acid; DHA, docosahexaenoic acid; 5,6-DHET, 5,6-dihydroxy-8,11,14-eicosatrienoic acid; 8,9-DHET, 8,9-dihydroxy-5,11,14-eicosatrienoic acid; 11,12-DHET, 11,12-dihydroxy-5,8,14-eicosatrienoic acid; 14,15-DHET, 14,15-dihydroxy-5,8,11-eicosatrienoic acid; 5,6-DiHETE, 5,6-dihydroxy-8,11,14,17-eicosatetraenoic acid; 17,18-DiHETE, 17,18-dihydroxy-5,8,11,14-eicosatetraenoic acid; 9,10-DiHOME, 9,10-dihydroxy-12-octadecenoic acid; 12,13-DiHOME, 12,13-dihydroxyoctadec-9-enoic acid; EPA, eicosapentaenoic acid; 9,10-EpOME, 9,10-epoxy-12-octadecenoic acid; 12,13-EpOME, 12,13-epoxy-9-octadecenoic acid; GCA, glycocholic acid; GCDCA, glycochenodeoxycholic acid; GDCA, glycodeoxycholic acid; GLCA, glycolithocholic acid; GUDCA, glycoursodeoxycholic acid; HCO, 7α-hydroxy-4-cholesten-3-one; HDCA, hyodeoxycholic acid; 14-HDoHE, 14-hydroxy docosahexaenoic acid; 12-HEPE, 12-hydroxy-5,8,10,14,17-eicosapentaenoic acid; 5-HETE, 5-hydroxy-6,8,11,14-eicosatetraenoic acid; 11-HETE, 11-hydroxy-5,8,12,14-eicosatetraenoic acid; 12-HETE, 12-hydroxy-5,8,10,14-eicosatetraenoic acid; 15-HETE, 15-hydroxy-5,8,11,13-eicosatetraenoic acid; 16-HETE, 16-hydroxy-5,8,11,14-eicosatetraenoic acid; 18-HETE, 18-hydroxy-5,8,11,14-eicosatetraenoic acid; 12-HHT, 12-hydroxy-5,8,10-heptadecatrienoic acid; 9-HODE, 9-hydroxy-10,12-octadecadienoic acid; 9-HOTrE, 9-hydroxy-10,12,15-octadecatrienoic acid; 9-HpODE, 9-hydroperoxyoctadeca-10,12-dienoic acid; 13-KODE, 13-keto-9,11,-octadecadienoic acid; LA, linoleic acid; α-LA, alpha-linoleic acid; LCA, lithocholic acid; PGD2, prostaglandin D2; PGE2, prostaglandin E2; SDS, sd score; TCA, taurocholic acid; TCDCA, taurochenodeoxycholic acid; TDCA, taurodeoxycholic acid; TUDCA, tauroursodeoxycholic acid; TXB2, thromboxane B2; UDCA, ursodesoxycholic acid.

**Fig 4:**
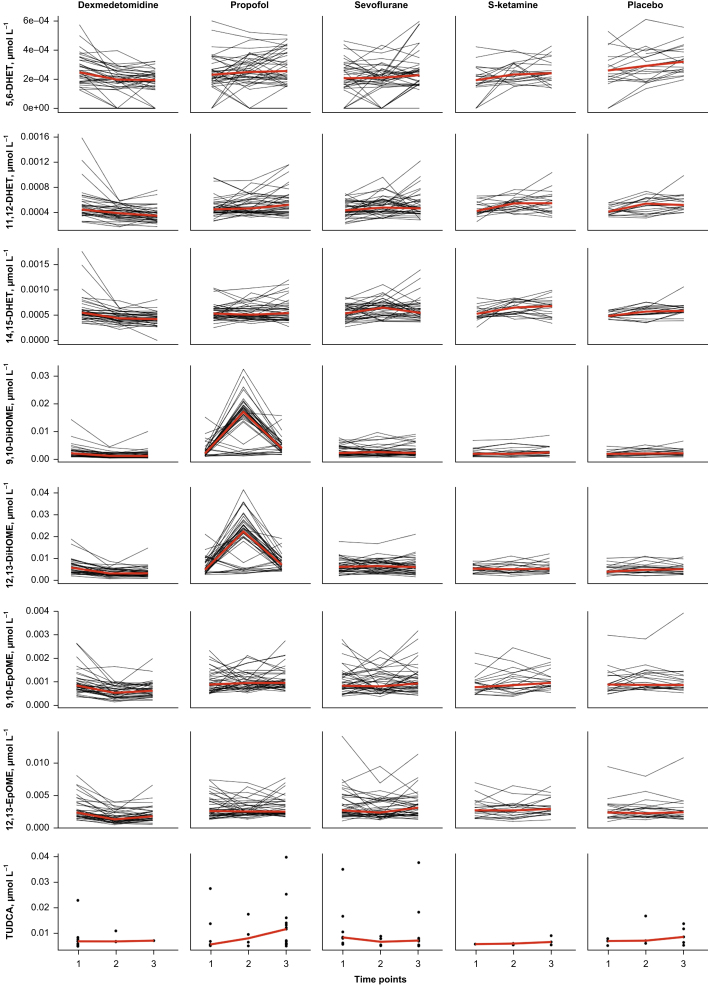
Line graphs of absolute concentrations of selected oxylipins and a point graph of TUDCA within each subject. Because of the number of values under the detection limit in bile acid TUDCA, a point graph was considered to be more informative for this metabolite. Red lines depict median values of each time point. 5,6-DHET, 5,6-dihydroxy-8,11,14-eicosatrienoic acid; 8,9-DHET, 8,9-dihydroxy-5,11,14-eicosatrienoic acid; 11,12-DHET, 11,12-dihydroxy-5,8,14-eicosatrienoic acid; 14,15-DHET, 14,15-dihydroxy-5,8,11-eicosatrienoic acid; 9,10-DiHOME, 9,10-dihydroxy-12-octadecenoic acid; 12,13-DiHOME, 12,13-dihydroxyoctadec-9-enoic acid; 9,10-EpOME, 9,10-epoxy-12-octadecenoic acid; 12,13-EpOME, 12,13-epoxy-9-octadecenoic acid; TUDCA, tauroursodeoxycholic acid.

Paired comparisons (time points 1 *vs* 2 or 1 *vs* 3) showed significant changes *vs* placebo in 62.5%, 12.5%, 5.0%, and 2.5% of the analytes in the dexmedetomidine, propofol, sevoflurane, and S-ketamine groups, respectively (Fig 2, Fig 3). Analyte concentrations within each subject at three time points are depicted in Figure 4. A summary of all significant changes in analytes in drug–placebo comparisons can be found in Table 1. Forest plots of all drug–drug comparisons, tables summarising all measured analytes, and absolute concentrations of significant analytes can be found in Supplementary Appendix B.

**Table 1:** Summary of significant oxylipin and bile acid changes *vs* placebo. Anaesthetic *vs* placebo comparisons of oxylipins and bile acids with a statistically significant change between time points 1 *vs* 2 or 1 *vs* 3, reported as standard deviation score (95% confidence interval), *P*-value. Changes between time points 1 *vs* 2 are represented if not otherwise stated; ∗1 *vs* 3 are reported, as statistical significance was not reached at 1 *vs* 2, yet change 1 *vs* 3 was significant. †Both time-point comparisons (1 *vs* 2 and 1 *vs* 3) to placebo were significant. ‡Time-point comparison 1 *vs* 2 was significant in all inter-drug comparisons. ¶ Time-point comparison 1 *vs* 3 was significant in all inter-drug comparisons. AA, arachidonic acid; CDCA, chenodeoxycholic acid; CYP, cytochrome P450; DCA, deoxycholic acid; 5,6-DHET, 5,6-dihydroxy-8,11,14-eicosatrienoic acid; 11,12-DHET, 11,12-dihydroxy-5,8,14-eicosatrienoic acid; 14,15-DHET, 14,15-dihydroxy-5,8,11-eicosatrienoic acid; 17,18-DiHETE, 17,18-dihydroxy-5,8,11,14-eicosatetraenoic acid; 9,10-DiHOME, 9,10-dihydroxy-12-octadecenoic acid; 12,13-DiHOME, 12,13-dihydroxyoctadec-9-enoic acid; EPA, eicosapentaenoic acid; 9,10-EpOME, 9,10-epoxy-12-octadecenoic acid; 12,13-EpOME, 12,13-epoxy-9-octadecenoic acid; GCA, glycocholic acid; GCDCA, glycochenodeoxycholic acid; GDCA, glycodeoxycholic acid; GLCA, glycolithocholic acid; GUDCA, glycoursodeoxycholic acid; HCO, 7α-hydroxy-4-cholesten-3-one; HDCA, hyodeoxycholic acid; 5-HETE, 5-hydroxy-6,8,11,14-eicosatetraenoic acid; 9-HODE, 9-hydroxy-10,12-octadecadienoic acid; 9-HOTrE, 9-hydroxy-10,12,15-octadecatrienoic acid; 13-KODE, 13-Oxo-9,11-octadecadienoic acid; LA, linoleic acid; α-LA, alpha-linoleic acid; LCA, lithocholic acid; LOX, lipoxygenase; TCDCA, taurochenodeoxycholic acid; TUDCA, tauroursodeoxycholic acid.

	Dexmedetomidine	Propofol	Sevoflurane	S-ketamine
Precursor for primary bile acid synthesis				
HCO	–0.71 (–1; –0.41), *P*=0.006∗			–0.76 (–1.11; –0.42), *P*=0.019∗
Primary bile acids				
CDCA	–0.46 (–0.67; –0.25), *P*=0.028			
Secondary bile acids				
DCA	–0.5 (–0.73; –0.27), *P*=0.031^†¶^			
LCA	–0.79 (–1.15; –0.43), *P*=0.019^†¶^			
Bile acid conjugates				
GCA	–0.93 (–1.35; –0.51), *P*=0.018^†^			
GCDCA	–1.46 (–1.91; –1.01), *P*<0.001^†^			
GDCA	–1.17 (–1.54; –0.8), *P*<0.001^†^			
GLCA	–1.15 (–1.65; –0.64), *P*=0.012^†¶^			
GUDCA	–1.21 (–1.61; –0.81), *P*<0.001^†^			
HDCA	–0.86 (–1.14; –0.58), *P*<0.001∗^¶^			
TCDCA	–1.68 (–2.31; –1.05), *P*<0.001∗			
TUDCA			–2.7 (–3.84; –1.55), *P*=0.015	
Oxylipin precursors				
AA	–1.46 (–1.95; –0.96), *P*<0.001∗^¶^			
EPA	–1.16 (–1.53; –0.79), *P*<0.001^†¶^	–0.81 (–1.18; –0.44), *P*=0.022		
CYP oxylipins from AA				
5,6-DHET	–1.23 (–1.76; –0.69), *P*=0.011∗			
11,12-DHET	–1.35 (–1.86; –0.83), *P*<0.001^†‡¶^			
14,15-DHET	–1.31 (–1.82; –0.8), *P*<0.001^†‡¶^			
CYP oxylipins from EPA				
17,18-DiHETE	–1.64 (–2.15; –1.13), *P*<0.001∗^¶^		1.18 (0.66; 1.7), *P*=0.01	
CYP oxylipins from LA				
9,10-DiHOME	–1.19 (–1.6; –0.78), *P*<0.001∗^¶^	2.29 (1.62; 2.96), *P*<0.001^‡^		
12,13-DiHOME	–1.22 (–1.66; –0.77), *P*<0.001∗^¶^	2.13 (1.42; 2.84), *P*<0.001^‡^		
9,10-EpOME	–1.39 (–1.96; –0.81), *P*=0.003^‡^			
12,13-EpOME	–1.25 (–1.74; –0.75), *P*=0.001			
LOX oxylipin from α-LA				
9-HOTrE	–1.27 (–1.84; –0.7), *P*=0.016^†¶^	1.98 (1.42; 2.53), *P*<0.001^‡^		
LOX oxylipin from AA				
5-HETE	–1.07 (–1.53; –0.61), *P*=0.008^†^			
LOX oxylipin from LA				
9-HODE	–1.32 (–1.79; –0.86), *P*<0.001^†‡¶^	1.35 (0.89; 1.82), *P*<0.001^‡^		
13-KODE	–1.2 (–1.73; –0.67), *P*=0.013∗^¶^			

Dexmedetomidine induced a widespread and significant decrease in both oxylipin and bile acids (Fig. 2 and Table 1). Amongst others, the CYP oxylipins 5,6-DHET –1.23 (–1.76; –0.69), *P*=0.011 (SDS [95% CI], *P*-value); 11,12-DHET –1.35 (–1.86; –0.83), *P*<0.001; 14,15-DHET –1.31 (–1.82; –0.8), *P*<0.001; 9,10-dihydroxyoctadecenoic acid (9,10-DiHOME) –1.19 (–1.6; –0.78), *P*<0.001; and 12,13-DiHOME –1.22 (–1.66; –0.77), *P*<0.001 were decreased (DiHOMEs at 1 *vs* 3). 13-Oxooctadeca-9,11-dienoic acid (13-KODE) decreased –1.20 (–1.73; –0.67), *P*=0.013 (1 *vs* 3). Of the significant changes observed *vs* placebo, 56% remained significant in all drug–drug comparisons (Table 1).

Propofol-induced changes focused on four oxylipins, and no changes were observed in bile acids (Fig. 2 and Table 1). CYP epoxygenase oxylipins from LA 9,10-DiHOME 2.29 (1.62; 2.96), *P*<0.001 and 12,13-DiHOME 2.13 (1.42; 2.84), *P*<0.001 were markedly increased, whilst no significant changes were observed in their less active precursors 9,10-epoxyoctadecenoic acid (EpOME) and 12,13-EpOME. LOX oxylipins from LA 9-hydroxyoctadecadienoic acid (9-HODE) and α-LA 9-hydroxyoctadecatrienoic acid (9-HOTrE) were increased, 1.35 (0.89; 1.82), *P*<0.001 and 1.98 (1.42; 2.53), *P*<0.001, respectively. EPA, an ω-3-fatty acid, was slightly decreased –0.81 (–1.18; –0.44), *P*=0.022. Of the significant changes observed *vs* placebo, the increases in 9,10-DiHOME, 12,13-DiHOME, 9-HODE, and 9-HOTrE were significant in all drug–drug comparisons (Table 1).

In the S-ketamine group, only a modest decrease in primary bile acid precursor 7α-hydroxy-cholestene-3-one (HCO) –0.76 (–1.11; –0.42), *P*=0.019 (1 *vs* 3) was observed (Fig. 3 and Table 1).

Sevoflurane decreased TUDCA –2.7 (–3.84; –1.55), *P*=0.015, a taurine conjugate of ursodeoxycholic acid (Fig. 3 and Table 1). Importantly, exact quantification of TUDCA was available for only 19.0% and 17.1% of the analysed samples in the placebo and sevoflurane groups, respectively (Fig. 4). An EPA-derived CYP epoxygenase oxylipin, 17,18-DiHETE, was slightly increased *vs* placebo, 1.18 (0.66; 1.7), *P*=0.01. This increase remained significant in comparison with dexmedetomidine.

The *in vitro* analysis of propofol emulsion dilution series showed that the levels of several oxylipins were strongly and dose-dependently increased in propofol emulsion containing plasma. With a focus on significantly increased oxylipins in the propofol group *in vivo*, 9,10-DiHOME and 12,13-DiHOME *in vitro* concentrations were 2.5- and 1.4-fold higher in 1:1000 v/v propofol emulsion dilution (mimicking relevant propofol dosing) *vs* the control plasma without propofol. In contrast, the levels of 9-HODE and 9-HOTrE were not elevated at relevant propofol dilution *vs* control plasma.

## Discussion

Dexmedetomidine, propofol, sevoflurane, and S-ketamine each induced unique changes in the measured analytes. Dexmedetomidine induced a wide-ranging decrease in bile acids and oxylipins. Contrary to dexmedetomidine, propofol induced a marked increase in DiHOME, which was most likely caused by the lipid emulsion of propofol. Only minor changes were observed in response to sevoflurane and S-ketamine. However, the observation of decreased TUDCA in response to sevoflurane may be of interest if verified in future studies.

Dexmedetomidine caused a wide-range reduction in oxylipins and bile acids (Table 1). The bile acid precursor HCO, primary bile acid chenodeoxycholic acid (CDCA), and several secondary bile acids and their conjugates were decreased. Changes in a single pathway seem insufficient to explain this global decrease. Possibly, a dexmedetomidine-induced reduction in hepatic blood flow could lead to a reduction in metabolites of hepatic origin. Dexmedetomidine has a substantial hepatic extraction ratio, and a decrease in cardiac output has been shown to reduce its rate of elimination.^12^ However, no significant reduction in liver blood flow could be demonstrated in dogs.^13^ In addition, vagal cholinergic stimulation is known to increase the rate of gall bladder emptying. Dexmedetomidine was shown to increase the discharge frequency of the vagal nerve and maintained acetylcholine levels during lipopolysaccharide (LPS) challenge in rodent models.^14^ ^15^ Possibly, dexmedetomidine-mediated vagal effects could result in a reduction of circulating bile acids.^16^

Dexmedetomidine decreased AA, CYP oxylipins from AA (5,6-DHET, 11,12-DHET, and 14,15-DHET), and the LOX oxylipin 5-HETE. DHETs are formed by the action of soluble epoxide hydroxylase (sEH) from epoxyeicosatrienoic acids (EETs), a conversion that attenuates the biological effects of EETs. Thus, a decrease in circulating DHET might reflect an increased effect of EETs. EETs protect against reperfusion injury in cardiomyocytes, possess anti-inflammatory properties, and inhibit nuclear factor kappa B (NF-κB) signalling.^3^ ^17^ ^18^ In a murine model, in response to inflammation induced by LPS, sEH inhibition led to decreases in DHETs.^19^ Surprisingly, in the same study, 5-HETE was decreased as well, suggesting cross-linked metabolism of AA oxylipins. Furthermore, the conversion of EpOME to DiHOME is governed by sEH. Upon LPS challenge, both DiHOMEs and DHET were lower in a murine model of sEH inhibition *vs* controls. EpOME and 5-HETE were unaffected, the decreases in 9- and 13-HODE (HODE, a precursor of 13-KODE) did not reach statistical significance, and a reduction in PGD2 was observed.^20^ The effects on PGD2 could not be determined in the current study because of an excessive number of zero values, although 9-HODE and 13-KODE were significantly decreased. To some extent, the changes we observed resemble those seen in sEH inhibition, which has been studied widely because of its possibly favourable cardiovascular and anti-inflammatory effects.^21^ Alternatively, CYP-mediated effects might contribute to reductions in DHET, EpOME, and DiHOME.^22^ ^23^ However, changes were observed in LOX metabolites as well. Further research is needed to uncover the mechanisms underlying the current observations.

Oxylipins possess vasoactive and pro- and anti-thrombotic properties. Dexmedetomidine decreased 9-HODE and 13-KODE (a derivative of 13-HODE). 9-HODE has pro-thrombotic properties in human saphenous vein endothelial cells, whilst previous research on the role of 13-HODE in thrombosis is inconclusive, suggesting both pro- and anti-thrombotic properties. In animal models, 13-HODE has been shown to cause vasorelaxation.^24^ It remains to be established whether these findings on 9-HODE and 13-KODE are physiologically relevant.

Dexmedetomidine induced 0.5- and 0.6-fold decreases (1 *vs* 3) in 9,10- and 12,13-DiHOME, respectively. This was preceded by a decrease in their precursor EpOMEs (1 *vs* 2). Initially, the search for molecular targets of mitochondrial toxicity in patients who have burn injuries and with acute respiratory distress syndrome (ARDS) led to the discovery of EpOME. However, further research suggested that the culprits were in fact the DiHOMEs, the toxic metabolites of EpOME.^2^ Toxic effects of DiHOMEs are mediated *via* mitochondrial dysfunction at high concentrations, and their administration in animal models caused mortality and histopathologic changes, suggesting ARDS.^25^ ^26^ Regardless of this association with ARDS and mitochondrial dysfunction, DiHOMEs have physiological roles at low concentrations.^27^ ^28^ It is worth considering whether reducing EpOMEs and DiHOMEs could be beneficial in patients with or at risk of ARDS. Indeed, dexmedetomidine has reduced inflammatory markers in animal models of acute lung injury, including myeloid differentiation primary response gene 88 (MyD88) and NF-κB.^29^ Interestingly, hyperactivation of the MyD88/NF-κB pathway is considered central to SARS-CoV-2-induced ARDS.^30^ Recently, in patients with or at risk of ARDS, sedation with dexmedetomidine was associated with significantly reduced in-hospital mortality in comparison with midazolam and propofol. This difference was thought to arise from dexmedetomidine-related reductions in inflammatory mediators, which is supported by the current results, along with lack of suppression of respiratory drive, reduced rate and shorter duration of delirium, and organoprotection.^31^

Moreover, vagal mechanisms *via* a cholinergic anti-inflammatory pathway (CAP) contribute to the hepatoprotection of dexmedetomidine *via* downregulation of Toll-like receptor 4 (TLR4)/MyD88/NF-κB.^32^ TLRs detect pathogen-associated molecular patterns. Briefly, activation of TLRs leads to elicited innate immune responses, including recruitment of MyD88 and activation of a transcription factor NF-κB, inducing pro-inflammatory cytokines, chemokines, and co-stimulatory molecules on dendritic cells. Because this cascade is essential for T-cell activation, inhibitors of TLR pathways might be beneficial in the termination of inflammation and in prevention of septic shock.^33^ Dexmedetomidine-induced organ protection has been associated with downregulation of MyD88/NF-κB.^34^ The current findings support this observation, as the reduction in DiHOMEs, EPOMEs, and 9-HODE and possibly increased EET (reflected in the current study by reduced DHET) would inhibit NF-κB pathway.^17^ ^35^ ^36^ Dexmedetomidine-mediated effects on CAP have been demonstrated to alleviate renal ischaemia–reperfusion injury and LPS-induced acute lung injury in rodent models, decreasing inflammatory mediators (amongst others interleukin [IL]-1β, IL-6, and tumour necrosis factor-alpha [TNF-α]). Disrupting this effect either by vagotomy, splenectomy, or agents antagonising the effects of dexmedetomidine on CAP abolished the observed effects.^14^, ^15^ It has been reported that in a cardiac cell line, administration of either 9,10- or 12,13-DiHOME resulted in massive release of TNF-α (and monocyte chemoattractant protein-1).^20^ Whether the observed reductions in oxylipins reflect the effect of dexmedetomidine on CAP seems an interesting possibility.

It has been suggested previously that anti-apoptotic properties of dexmedetomidine could also lead to undesired effects. In a rodent model, dexmedetomidine promoted metastasis in breast, lung, and colon cancers; further mechanistic translational studies were encouraged to understand these observations.^37^ Indeed, dexmedetomidine enhanced cancer cell proliferation and migration by upregulating anti-apoptotic proteins in human lung and neuroglioma cell lines *in vitro*.^38^ Recently, the effects of sEH deletion were studied in a rodent model of breast cancer; increased angiogenesis, tumour growth, and altered tumour oxylipin profile were reported. AA oxylipins 8,9-, 11,12-, and 14,15-DHET decreased, and the corresponding EETs were unaffected. In addition, 9,10- and 11,13-EpOMEs were increased, and increases in corresponding DiHOME were non-significant. Moreover, 17,18-DiHETE was increased.^39^ Albeit similar findings on DHET were observed in the dexmedetomidine group, contrasting decreases in 9,10- and 12,13-EpOME and DiHOME and 17,18-DiHETE were observed in the current study. However, many of the oxylipins have the potential to inhibit NF-κB, which has a complex role in malignancy, and is often constitutively active in malignant cells and the tumour microenvironment.^40^ Further research might offer answers to the role of oxylipins in this context.

In contrast to dexmedetomidine, propofol substantially increased LA derivatives 9,10- and 12,13-DiHOME. Their concentrations peaked at 17.1 (9.5; 19.4) and 22.4 (13.1; 25.1) nmol L^−1^ in the propofol group, and corresponding values in the placebo group were 2.2 (1.8; 3.6) and 4.9 (3.8; 6.6) nmol L^−1^ (Supplementary Appendix B). These changes in the propofol group were 7.6- and 4.5-fold from baseline (1 *vs* 2), respectively. In comparison, 9,10- and 12,13-DiHOME concentrations, mean (sd), in serial measurements of six patients hospitalised with severe SARS-CoV-2 were 56.4 (87.3) and 71.8 (113.7) nmol L^−1^ and in mouse burn models 93.2 (33.8) and 292.5 (122.8) nmol L^−1^, respectively.^41^ ^42^ *In vitro* mitochondrial dysfunction was induced by 9,10-DiHOME at 180 μmol L^−1^.^26^ In the current study, increases were also observed in other LA and α-LA derivatives (LOX oxylipins 9-HODE and 9-HOTrE, respectively). All the aforementioned oxylipins were lowered by dexmedetomidine. A likely cause for increased DiHOME is the lipid emulsion of propofol, as suggested by the *in vitro* analysis. Consistent with this, an increase in 12,13-DiHOME has been demonstrated in response to Intralipid, an often-used proxy for lipid emulsion of propofol.^43^

The combined effects of propofol and DiHOMEs might prove interesting concerning cellular bioenergetics. Especially during catabolic states, fatty acids are broken down in mitochondrial fatty acid oxidation (FAO) to yield energy. To access the mitochondria, both LA and α-LA require carnitine transport.^44^ This transport was inhibited by propofol in animal models, and inhibition also occurred in a rare case of propofol infusion syndrome.^45^ *In vitro*, clinically relevant concentrations of propofol directly inhibited FAO and mitochondrial respiration in human heart and skeletal muscle.^46^ Physiologically, elevated 12,13-DiHOME in response to exercise increased fatty acid uptake to skeletal and cardiac myocytes, increasing FAO.^28^ In high concentrations, DiHOMEs induce mitochondrial dysfunction.^2^ ^3^ ^26^ Interestingly, myocardial and skeletal muscle fat accumulation has been observed in a *post-mortem* case report of propofol infusion syndrome.^47^ Proposed pathophysiological mechanisms for propofol infusion syndrome include mitochondrial dysfunction and inhibition of FAO.^45^ In the current study, we observed that brief propofol sedation markedly elevated circulating DiHOMEs. In light of previous literature on bioenergetic effects of both propofol and DiHOMEs, the behaviour of DiHOMEs during prolonged propofol infusions in clinical practise should be established.

Moreover, DiHOMEs possess immunomodulatory capabilities. DiHOMEs are synthesised by activated neutrophils and induce neutrophil chemotaxis (in the concentration of ∼10 nM), and their esters suppress the neutrophil respiratory burst mechanism *in vitro* (20–200 μM).^2^ ^48^ Interestingly, previous research in animal models has suggested increased susceptibility to bacterial infection associated with propofol administration.^49^ Propofol-induced suppression of neutrophil respiratory burst in comparison with isoflurane has been described.^50^

Although there was a high number of missing values, administration of sevoflurane resulted in a statistically significant reduction in TUDCA, a secondary bile acid with neuroprotective effects in neurodegenerative disease and ischaemic stroke.^5^ In a neonatal animal model, cognitive impairment and hippocampal endoplasmic reticulum stress induced by repeated sevoflurane exposure were ameliorated by administration of TUDCA.^6^ Sevoflurane-induced effects on TUDCA remain uncertain and need to be verified in future studies.

Oxylipins and bile acids were relatively unaffected by S-ketamine regardless of previous findings on glucose, lactate, and amino-acid metabolites.^4^ In our study, only a decrease in primary bile acid precursor HCO was observed. As no changes in bile acids were observed, the finding is likely of no clinical relevance.

A few limitations of our study can be addressed. As an explorative study on healthy subjects, our ability to assess the clinical impact of the observed changes remains limited, and further research is needed. Only male subjects were included because of the subsequent positron emission tomography study of human consciousness. Brief anaesthetic or sedative exposure and EC_50_ doses result in smaller exposure in comparison with clinical practice. In previous studies, lipid emulsions such as Intralipid have been used as a proxy for the lipid emulsion of propofol. As no control group for propofol free lipid emulsion was available, the effects of propofol and the formulation cannot be differentiated. As oxylipins are derived from dietary PUFA (LA, α-LA, AA, and EPA), it is possible that long-term dietary tendencies affect oxylipin levels. However, the current study was conducted on fasted subjects, and therefore, the possible confounding effect of PUFA-rich meals on the results was minimised. As an overall limitation, this study was limited to targeted metabolomics. Lastly, because of a relatively high number of missing values in TUDCA, further research is needed to verify the observed effect.

In conclusion, in this exploratory study, we observed that a 1 h administration of dexmedetomidine, propofol, sevoflurane, or S-ketamine causes acute, biologically interesting changes in oxylipin and bile acid profiles of healthy male subjects. Dexmedetomidine induced a broad decrease in oxylipins and bile acids. The observations on oxylipins might be connected to proposed organ-protective effects of dexmedetomidine. Propofol-induced increase in DIHOMEs is likely attributable to the lipid emulsion of propofol. In high concentrations, DiHOMEs are associated with ARDS and mitochondrial dysfunction. In contrast, dexmedetomidine induced a gradual decrease in DiHOMEs. Further research is needed to establish the behaviour of DiHOME in the context of prolonged propofol or dexmedetomidine administration. Data suggest a sevoflurane-induced reduction in TUDCA, but the observation needs to be verified by future studies.

## Authors' contributions

Study design/planning: HS, TL, AR, MN, MP, AN

Subject recruitment: LL

Experiment conduct: LL, AS, KK, TL, KV

Data analysis: TV, AN

Laboratory analysis: MNe, MNi

Writing of article: AN

Revising of article: all authors
