# Supplementary material for: Circulating oxylipin and bile acid profiles of dexmedetomidine, propofol, sevoflurane, and S-ketamine: a randomised controlled trial using tandem mass spectrometry
Source: BJA Open. 2022 Dec 12;4:100114. doi: 10.1016/j.bjao.2022.100114 (PMC10430865; doi:10.1016/j.bjao.2022.100114)
Supplement: Supplementary Appendix A [file mmc1.docx]

**Appendix A, supplementary methods, lipidomics analysis**

The concentrations of plasma bile acids and lipid mediators were analyzed at the Department of Clinical Pharmacology, University of Helsinki, using a Nexera X2 UHPLC system (Shimadzu, Kyoto, Japan) coupled to a 5500 Qtrap mass spectrometer interfaced with an electrospray ion source (ABSciex, Toronto, ON). Reference bile acids and lipid mediators, and the stable isotope labeled internal standards were purchased from Cayman Chemical Company (Ann Arbor, MI), except for hyodeoxycholic acid D5 (HDCA-D5), glycoursodeoxycholic acid D5 (GUDCA-D5), lithocholic acid D4 (LCA-D4), tauroursodeoxycholic acid D5 (TUDCA-D5), taurochenodeoxycholic acid D5 (TCDCA-D5), taurocholic acid D5 (TCA-D5), taurodeoxycholic acid D5 (TDCA-D5), taurolithocholic acid D5 (TLCA-D5), and 7α-Hydroxy-4-cholesten-3-one D7, which were purchased from Toronto Research Chemicals (North York, ON). Other chemicals and organic solvents were of commercially available analytical grade.

A total of 17 bile acids were quantified as previously described with minor modifications^1^. Calibration standards (0.001-5 µmol/L) and quality controls for each bile acid, prepared in charcoal stripped plasma, were processed along with the plasma samples. The plasma samples (150 μL) were mixed with 50 μL of 0.05% formic acid containing the internal standards, and the sample mixtures were allowed to incubate at +4 ˚C for 10 minutes. Plasma bile acids were extracted using a Strata-X polymeric reversed-phase 96-well extraction plate (10 mg/well, Phenomenex, Torrance, CA), which was pre-conditioned according to manufacturer’s instructions prior to sample loading. The wells were washed with 100 μL of 0.05% formic acid and 100 μL of 5% methanol, and the analytes were eluted with 100 μL of methanol followed by 100 μL of acetonitrile. The sample extracts were dried using a vacuum evaporator (GeneVac, Thermo Fisher Scientific, Waltham, MA, USA) and reconstituted in 100 μL of 60% methanol. A sample volume of 5 µL was injected into the LC-MS system. The chromatographic separation was achieved on Atlantis T3 analytical column, 2.1×100 mm, 3 μm particle size (Waters Corp., Milford, MA, USA) using the gradient elution of 5 mM ammonium acetate containing 0.005% formic acid in water (mobile phase A) and methanol (mobile phase B). The flow rate and the column temperature were set at 0.3 mL/min and 40 ˚C. The mass spectrometer was operated in negative multiple reaction monitoring (MRM) mode, except for 7-OH-4-cholesten-3-one, which was analyzed in positive mode (ion transition m/z 401-177). The within-day and between-day accuracies (% of nominal concentration) of the method ranged from 87.9% to 113%, and precisions (CV%) were ≤ 14.1%, except for LLOQ for which accuracies and precisions were ±20 and <20% (Supplementary Table 1).

A total of 158 target eicosanoids and related compounds were monitored in human plasma as previously described using the authentic reference standards as calibrators ^2^. Prior to analysis, the aliquots of 100 µL plasma were precipitated with 400 µL of methanol containing 18 stably labeled internal standards. The sample mixtures were centrifuged for 10 min at 14000 g, and the supernatants were diluted with 0.03% formic acid (1:5 v/v) before loading into the pre-conditioned Strata-X extraction plate (10 mg/well, Phenomenex). The extraction wells were serially washed with 100 µL of 0.03% formic acid and 100 µL of 10% methanol, and eluted with 150 µL of methanol followed by 150 µl of ethanol. The eluent was evaporated to dryness in vacuum evaporator (Thermo Fisher Scientific) and the lipids were reconstituted in 25 µL of methanol. The chromatographic separation of lipids was performed on a reversed-phase analytical column Kinetex C8 2.1×150 mm, 2.6 µM particle size (Phenomenex) utilizing 0.1 % formic acid and acetonitrile as mobile phase A and B. The mobile phase flow rate and the column temperature were set at 0.35 mL/min and 40 ˚C. The mass spectrometer was operated both in positive and in negative MRM mode applying the scheduled MRM method to minimize the number of ion transitions acquired simultaneously. A signal-to-noise (S/N) ratio larger than 3 was defined as the detection limit and the S/N ratio larger than 7 was set as the quantification limit. Twenty-eight lipids were reliably quantified in plasma. The within-day and between-day accuracy and precision of the method was determined by 3-6 different concentration levels of quality control samples covering the relevant plasma concentration range for each oxylipin. The accuracies were within ±15% and the precisions below 15% at the QC concentration levels of at least three times of LLOQ, and <20% and ±20 at the level of LLOQ (Supplementary Table 1). All analytes were quantified using internal standard methods.

1. Xiang X, Han Y, Neuvonen M, Laitila J, Neuvonen PJ, Niemi M. High performance liquid chromatography-tandem mass spectrometry for the determination of bile acid concentrations in human plasma. *J Chromatogr B Anal Technol Biomed Life Sci* Elsevier; 2010; **878**: 51–60

2. Yamada M, Kita Y, Kohira T, et al. A comprehensive quantification method for eicosanoids and related compounds by using liquid chromatography/mass spectrometry with high speed continuous ionization polarity switching. *J Chromatogr B Anal Technol Biomed Life Sci* Elsevier; 2015; **995**–**996**: 74–84

**Appendix A, supplementary Table 1.**

Within-day and between-day precision and accuracy (n=6), lower limit of quantification (LLOQ) and internal standard (IS) used for each analyte.

|  |  | Within-day | | | | Between-day | | | |  |
| --- | --- | --- | --- | --- | --- | --- | --- | --- | --- | --- |
|  |  | Low concentration | | High concentration | | Low concentration | | High concentration | |  |
|  | LLOQ | Precision | Accuracy | Precision | Accuracy | Precision | Accuracy | Precision | Accuracy | IS |
|  | [nM] | CV% [nM] | (%) | CV% [nM] | (%) | CV% [nM] | (%) | CV% [nM] | (%) |  |
| Precursor for bile acid synthesis |  |  |  |  |  |  |  |  |  |  |
| HCO (7α-Hydroxy-4-cholesten-3-one) | 5.0 | 8.0 [50] | 97.1 | 3.4 [1000] | 94.1 | 4.6 [50] | 95.8 | 3.0 [1000] | 103 | 7α-Hydroxy-4-cholesten-3-one-D7 |
| Primary bile acids |  |  |  |  |  |  |  |  |  |  |
| CA (Cholic acid) | 5.0 | 3.3 [25] | 111 | 2.2 [1000] | 99.1 | 7.8 [25] | 108 | 5.0 [1000] | 92.1 | CA-D4 |
| CDCA (Chenodeoxycholic acid) | 5.0 | 5.5 [25] | 106 | 7.1 [1000] | 99.4 | 6.0 [25] | 96.2 | 4.9 [1000] | 99.4 | CDCA-D4 |
| Secondary bile cids |  |  |  |  |  |  |  |  |  |  |
| DCA (Deoxycholic acid) | 1.0 | 3.3 [5] | 113 | 4.0 [500] | 89.7 | 7.3 [5] | 105 | 2.3 [500] | 102 | DCA-D4 |
| LCA (Lithocholic acid) | 1.0 | 5.5 [5] | 102 | 3.3 [500] | 97.7 | 7.2 [5] | 103 | 8.7 [500] | 103 | LCA-D4 |
| UDCA (Ursodesoxycholic acid) | 5.0 | 3.6 [25] | 104 | 2.9 [500] | 91.7 | 5.3 [25] | 96.5 | 4.9 [500] | 103 | UDCA-D4 |
| Bile acid conjugates |  |  |  |  |  |  |  |  |  |  |
| GCA (Glycocholic acid) | 5.0 | 3.1 [25] | 98.8 | 3.0 1000] | 95.1 | 4.2 [25] | 96.1 | 5.2 [1000] | 95.7 | GCA-D4 |
| GCDCA (Glycochenodeoxycholic acid) | 1.0 | 3.8 [5] | 99.8 | 1.6 [1000] | 97.6 | 5.7 [5] | 106 | 6.1 [1000] | 87.7 | GCDCA-D4 |
| GDCA (Glycodeoxycholic acid) | 1.0 | 9.0 [5] | 94.4 | 2.5 [500] | 96.5 | 12.2 [5] | 101 | 5.2 [1000] | 95.6 | GDCA-D5 |
| GLCA (Glycolithocholic acid) | 1.0 | 8.1 [5] | 87.9 | 14.1 [500] | 94.4 | 11.6 [5] | 99.0 | 5.8 [500] | 101 | CDCA-D4 |
| GUDCA (Glycoursodeoxycholic acid) | 5.0 | 2.4 [25] | 98.7 | 3.0 [500] | 92.4 | 5.3 [25] | 107 | 7.2 [500] | 101 | GUDCA-D5 |
| HDCA (Hyodeoxycholic acid) | 5.0 | 4.3 [25] | 103 | 3.7 [500] | 95.2 | 4.5 [25] | 97.2 | 7.9 [500] | 102 | HDCA-D5 |
| TCA (Taurocholic acid) | 5.0 | 7.0 [25] | 105 | 2.1 [1000] | 104 | 8.2 [25] | 101 | 7.5 [1000] | 96.2 | TCA-D5 |
| TCDCA (Taurochenodeoxycholic acid) | 5.0 | 5.2 [25] | 108 | 2.3 [500] | 100 | 3.3 [25] | 108 | 6.0 [500] | 106 | TCDCA-D5 |
| TDCA (Taurodeoxycholic acid) | 5.0 | 5.0 [25] | 98.8 | 3.3 [500] | 94.0 | 6.5 [25] | 96.5 | 6.0 [500] | 94.7 | TDCA-D5 |
| TLCA (Taurolithocholic acid) | 5.0 | 4.1 [25] | 96.2 | 2.8 [1000] | 94.6 | 7.4 [25] | 108 | 2.9 [1000] | 94.8 | TLCA-D5 |
| TUDCA (Tauroursodeoxycholic acid) | 5.0 | 3.5 [25] | 102 | 3.2 [500] | 100 | 3.8 [25] | 98.7 | 3.8 [500] | 94.6 | TUDCA-D5 |
| Oxylipin precursors |  |  |  |  |  |  |  |  |  |  |
| AA (Arachidonic acid) | 40 | 3.1 [300] | 98.2 | 2.9 [3000] | 97.2 | 3.6 [300] | 99.0 | 1.9 [3000] | 97.4 | AA-D8 |
| EPA (Eicosapentaeonic acid) | 40 | 2.2 [80] | 103 | 4.0 [600] | 95.1 | 6.7 [80] | 98.0 | 2.4 [600] | 97.0 | EPA-D5 |
| COX oxylipins from AA |  |  |  |  |  |  |  |  |  |  |
| 12-HHT (12-hydroxy-5,8,10-heptadecatrienoic acid) | 0.2 | 9.7 [0.8] | 105 | 5.9 [30] | 102 | 5.7 [0.8] | 105 | 3.2 [30] | 103 | LTB4 D4 |
| PGD2 (Prostaglandin D2) | 0.5 | 10.7 [0.8] | 108 | 2.9 [30] | 99.8 | 1.9 [0.8] | 110 | 5.2 [30] | 107 | PGD2-D4 |
| PGE2 (Prostaglandin E2) | 0.2 | 5.0 [0.8] | 111 | 4.0 [30] | 101 | 5.3 [0.8] | 106 | 3.3 [30] | 105 | PGF2a-D4 |
| TXB2 (Thromboxane B2) | 1.0 | 3.5 [1.5] | 105 | 6.0 [30] | 96.1 | 4.3 [1.5] | 108 | 2.8 [30] | 105 | TXB2-D4 |
| CYP oxylipins from AA |  |  |  |  |  |  |  |  |  |  |
| 5,6-DHET (5,6-dihydroxy-8,11,14-eicosatrienoic acid) | 0.1 | 3.8 [0.8] | 110 | 4.1 [30] | 92.7 | 5.4 [0.8] | 112 | 6.8 [30] | 94.6 | LTB4 D4 |
| 8,9-DHET (8,9-dihydroxy-5,11,14-eicosatrienoic acid) | 0.3 | 6.8 [0.8] | 110 | 3.0 [30] | 103 | 6.8 [0.8] | 103 | 5.5 [30] | 101 | LTB4 D4 |
| 11,12-DHET (11,12-dihydroxy-5,8,14-eicosatrienoic acid) | 0.1 | 2.8 [0.8] | 111 | 3.0 [30] | 99.6 | 4.2 [0.8] | 110 | 7.0 [30] | 96.3 | LTB4 D4 |
| 14,15-DHET (14,15-dihydroxy-5,8,11-eicosatrienoic acid) | 0.1 | 7.8 [0.8] | 105 | 3.0 (30] | 101 | 7.4 [0.8] | 106 | 4.5 [30] | 100 | LTB4 D4 |
| 16-HETE (16-hydroxy-5,8,11,14-eicosatetraenoic acid) | 0.2 | 6.9 [0.8] | 99.8 | 4.2 [30] | 98.7 | 10.0 [0.8] | 95.0 | 8.2 [30] | 104 | 15-HETE-D8 |
| 18-HETE (18-Hydroxy-5,8,11,14-eicosatetraenoic acid) | 0.2 | 13.6 [0.8] | 101 | 3.8 [30] | 106 | 9.2 [0.8] | 94.9 | 7.5 [30] | 103 | 15-HETE-D8 |
| 20-COOH-AA (5,8,11,14-eicosatetraenedioic acid) | 1.0 | 10.3 [1.5] | 95.3 | 4.1 [30] | 103 | 9.1 [1.5] | 99.4 | 6.6 [30] | 102 | LTB4 D4 |
| CYP oxylipins from EPA |  |  |  |  |  |  |  |  |  |  |
| 5,6-DiHETE (5,6-dihydroxy-8,11,14,17-eicosatetraenoic acid) | 8.0 | - | - | 9.5 [30] | 105 | - | - | 8.5 (30) | 105 | LTB4 D4 |
| 17,18-DiHETE (17,18-dihydroxy-5,8,11,14-eicosatetraenoic acid) | 3.0 | 5.9 [6.0] | 98.4 | 5.6 [30] | 107 | 11.6 [6.0] | 100 | 11.4 [30] | 106 | LTB4 D4 |
| CYP oxylipins from LA |  |  |  |  |  |  |  |  |  |  |
| 9,10-DiHOME (9,10-dihydroxy-12-octadecenoic acid) | 0.1 | 5.2 [0.8] | 107 | 3.3 [30] | 96.7 | 3.2 [0.8] | 109 | 3.0 [30] | 102 | LTB4 D4 |
| 12,13-DiHOME (12,13-Dihydroxyoctadec-9-enoic acid) | 0.3 | 6.9 [0.8] | 115 | 2.6 [30] | 98.8 | 6.3 [0.8] | 106 | 4.6 [30] | 105 | LTB4 D4 |
| 9,10-EpOME (9,10-epoxy-12-octadecenoic acid) | 0.1 | 7.5 [0.8] | 107 | 6.7 [30] | 97.5 | 7.4 [0.8] | 106 | 4.5 [30] | 100 | 5-HETE-D8 |
| 12,13-EpOME (12,13-epoxy-9-octadecenoic acid) | 0.3 | 13.2 [0.8] | 105 | 2.9 [30] | 94.7 | 11.2 [0.8] | 103 | 9.2 [30] | 93.0 | 5-HETE-D8 |
| LOX oxylipin from alpha-LA |  |  |  |  |  |  |  |  |  |  |
| 9-HOTrE (9-hydroxy-10,12,15-octadecatrienoic acid) | 0.2 | 7.5 [0.8] | 107 | 2.1 [30] | 101 | 5.8 [0.8] | 96.6 | 6.1 [30] | 99.8 | LTB4 D4 |
| LOX oxylipins from AA |  |  |  |  |  |  |  |  |  |  |
| 5-HETE (5-hydroxy-6,8,11,14-eicosatetraenoic acid) | 0.1 | 5.4 [0.8] | 104 | 3.9 [30] | 98.4 | 9.9 [0.8] | 97.2 | 5.7 [30] | 97.3 | 5-HETE-D8 |
| 11-HETE (11-hydroxy-5,8,12,14-eicosatetraenoic acid) | 0.1 | 8.9 [0.8] | 100 | 5.2 [30] | 102 | 6.5 [0.8] | 102 | 7.0 [30] | 96.4 | 12-HETE-D8 |
| 12-HETE (12-hydroxy-5,8,10,14-eicosatetraenoic acid) | 0.2 | 14.4 [0.8] | 95.6 | 2.3 [30] | 95.3 | 14.9 [0.8] | 92.0 | 6.2 [30] | 106 | 12-HETE-D8 |
| 15-HETE (15-hydroxy-5,8,11,13-eicosatetraenoic acid) | 0.5 | 7.8 [0.8] | 102 | 2.7 [30] | 94.7 | 9.4 [0.8] | 105 | 10.6 [30] | 99.6 | 15-HETE-D8 |
| LOX oxylipin from DHA |  |  |  |  |  |  |  |  |  |  |
| 14-HDoHE (14-hydroxy docosahexaenoic Acid) | 0.5 | 6.3 [0.8] | 103 | 5.4 [30] | 101 | 12.1 [0.8] | 94.9 | 8.2 [30] | 102 | 12-HETE-D8 |
| LOX oxylipin from DHA |  |  |  |  |  |  |  |  |  |  |
| 12-HEPE (12-hydroxy-5,8,10,14,17-eicosapentaenoic acid) | 0.3 | 4.0 [0.8] | 97.3 | 3.4 [30] | 97.1 | 7.4 [0.8] | 95.7 | 6.9 [30] | 103 | 15-HETE-D8 |
| LOX oxylipins from LA |  |  |  |  |  |  |  |  |  |  |
| 9-HODE (9-hydroxy-10,12-octadecadienoic acid) | 0.5 | 15.7 [0.8] | 92.3 | 3.0 [30] | 93.1 | 12.2 [0.8] | 92.4 | 5.3 30] | 99.9 | 15-HETE-D8 |
| 13-KODE (13-keto-9,11,-octadecadienoic acid) | 0.5 | 8.1 [0.8] | 108 | 4.8 [30] | 102 | 8.9 [0.8] | 93.4 | 9.4 [30] | 103 | 15-HETE-D8 |
