# Supplementary material for: Circulating oxylipin and bile acid profiles of dexmedetomidine, propofol, sevoflurane, and S-ketamine: a randomised controlled trial using tandem mass spectrometry
Source: BJA Open. 2022 Dec 12;4:100114. doi: 10.1016/j.bjao.2022.100114 (PMC10430865; doi:10.1016/j.bjao.2022.100114)
Supplement: Supplementary Appendix B [file mmc2.docx]

**Appendix B, supplementary Table 2. All analytes and summary of significant changes *vs.* placebo**

|  |  | **Dex** | **Pro** | **Sev** | **Ket** |
| --- | --- | --- | --- | --- | --- |
|  |  |  |  |  |  |
| **Precursor for bile acid synthesis** |  |  |  |  |  |
| HCO (7α-Hydroxy-4-cholesten-3-one) |  | (↓) |  |  | (↓) |
|  |  |  |  |  |  |
| **Primary bile acids** |  |  |  |  |  |
| CA (cholic acid) |  |  |  |  |  |
| CDCA (chenodeoxycholic acid) |  | ↓ |  |  |  |
|  |  |  |  |  |  |
| **Secondary bile cids** |  |  |  |  |  |
| DCA (deoxycholic acid) |  | ↓ |  |  |  |
| LCA (lithocholic acid) |  | ↓ |  |  |  |
| UDCA (ursodesoxycholic acid) |  |  |  |  |  |
|  |  |  |  |  |  |
| **Bile acid conjugates** |  |  |  |  |  |
| GCA (glycocholic acid) |  | ↓ |  |  |  |
| GCDCA (glycochenodeoxycholic acid) |  | ↓↓ |  |  |  |
| GDCA (glycodeoxycholic acid) |  | ↓↓ |  |  |  |
| GLCA (glycolithocholic acid) |  | ↓↓ |  |  |  |
| GUDCA (glycoursodeoxycholic acid) |  | ↓↓ |  |  |  |
| HDCA (hyodeoxycholic acid) |  | (↓) |  |  |  |
| TCA (taurocholic acid) |  |  |  |  |  |
| TCDCA (taurochenodeoxycholic acid) |  | (↓↓) |  |  |  |
| TDCA (taurodeoxycholic acid) |  |  |  |  |  |
| TUDCA (tauroursodeoxycholic acid) |  |  |  | ↓↓↓ |  |
|  |  |  |  |  |  |
| **Oxylipin precursors** |  |  |  |  |  |
| AA (arachidonic acid) |  | (↓↓) |  |  |  |
| EPA (eicosapentaeonic acid) |  | ↓↓ | ↓ |  |  |
|  |  |  |  |  |  |
| **COX oxylipins from AA** |  |  |  |  |  |
| 12-HHT (12-hydroxy-5,8,10-heptadecatrienoic acid) | |  |  |  |  |
| TXB2 (thromboxane B2) |  |  |  |  |  |
|  |  |  |  |  |  |
| **CYP oxylipins from AA** |  |  |  |  |  |
| 5,6 -DHET (5,6-dihydroxy-8,11,14-eicosatrienoic acid) | | (↓↓) |  |  |  |
| 11,12-DHET (11,12-dihydroxy-5,8,14-eicosatrienoic acid) | | ↓↓ |  |  |  |
| 14,15-DHET (14,15-dihydroxy-5,8,11-eicosatrienoic acid) | | ↓↓ |  |  |  |
| 16-HETE (16-hydroxy-5,8,11,14-eicosatetraenoic acid) | |  |  |  |  |
| 18-HETE (18-hydroxy-5,8,11,14-eicosatetraenoic acid) | |  |  |  |  |
| 20-COOH-AA (5,8,11,14-eicosatetraenedioic acid) | |  |  |  |  |
|  |  |  |  |  |  |
| **CYP oxylipins from EPA** |  |  |  |  |  |
| 17,18-DiHETE (17,18-dihydroxy-5,8,11,14-eicosatetraenoic acid) | | (↓↓) |  | ↑↑ |  |
|  |  |  |  |  |  |
| **CYP oxylipins from LA** |  |  |  |  |  |
| 9,10- DiHOME (9,10-dihydroxy-12-octadecenoic acid) | | (↓↓) | ↑↑↑ |  |  |
| 12,13-DiHOME (12,13-dihydroxyoctadec-9-enoic acid) | | (↓↓) | ↑↑↑ |  |  |
| 9,10-EpOME (9,10-epoxy-12-octadecenoic acid) | | ↓↓ |  |  |  |
| 12,13-EpOME (12,13-epoxy-9-octadecenoic acid) | | ↓↓ |  |  |  |
|  |  |  |  |  |  |
| **LOX oxylipin from alpha-LA** |  |  |  |  |  |
| 9-HOTrE (9-hydroxy-10,12,15-octadecatrienoic acid) | | ↓↓ | ↑↑ |  |  |
|  |  |  |  |  |  |
| **LOX oxylipins from AA** |  |  |  |  |  |
| 5-HETE (5-hydroxy-6,8,11,14-eicosatetraenoic acid) | | ↓↓ |  |  |  |
| 11-HETE (11-hydroxy-5,8,12,14-eicosatetraenoic acid) | |  |  |  |  |
| 12-HETE (12-hydroxy-5,8,10,14-eicosatetraenoic acid) | |  |  |  |  |
| 15-HETE (15-hydroxy-5,8,11,13-eicosatetraenoic acid) | |  |  |  |  |
|  |  |  |  |  |  |
| **LOX oxylipin from DHA** |  |  |  |  |  |
| 14-HDoHE (14-hydroxy docosahexaenoic acid) |  |  |  |  |  |
|  |  |  |  |  |  |
| **LOX oxylipin from DHA** |  |  |  |  |  |
| 12-HEPE (12-hydroxy-5,8,10,14,17-eicosapentaenoic acid) | |  |  |  |  |
|  |  |  |  |  |  |
| **LOX oxylipins from LA** |  |  |  |  |  |
| 9-HODE (9-hydroxy-10,12-octadecadienoic acid) | | ↓↓ | ↑↑ |  |  |
| 13-KODE (13-keto-9,11,-octadecadienoic acid) |  | (↓↓) |  |  |  |

All measured analytes, abbreviations, and names. Statistically significant change *vs.* placebo at timepoints 1 *vs.* 2 are included and represented with arrows, and for those markers that did not reach statistical significance at timepoints 1 *vs.* 2 the arrows in brackets symbolise significant change in timepoints 1 *vs.* 3. Direction of the arrow depicts direction of change (decrease *vs.* increase). Number of arrows represents the magnitude of change (1 arrow absolute change 0-1 SDS, 2 arrows 1-2 SDS, 3 arrows 2-3 SDS).

**Appendix B, supplementary Table 3.** Absolute analyte concentrations, dexmedetomidine *vs*. placebo.

|  | **Dexmedetomidine** | | | **Placebo** | | |
| --- | --- | --- | --- | --- | --- | --- |
| **Precursor for primary bile acid synthesis** | **Timepoint 1** | **Timepoint 2** | **Timepoint 3** | **Timepoint 1** | **Timepoint 2** | **Timepoint 3** |
| **HCO** (7α-Hydroxy-4-cholesten-3-one) | 0.042 (0.027; 0.067) | 0.034 (0.023; 0.051) | **0.029 (0.019; 0.044)** | 0.033 (0.018; 0.051) | 0.028 (0.016; 0.037) | 0.032 (0.022; 0.048) |
| **Primary bile acids** | | | | | | |
| **CDCA** (chenodeoxycholic acid) | 0.125 (0.037; 0.175) | **0.036 (0.015; 0.063)** | 0.022 (0.013; 0.042) | 0.039 (0.019; 0.092) | 0.018 (0.013; 0.044) | 0.021 (0.014; 0.046) |
| **Secondary bile acids** | | | | | | |
| **DCA** (deoxycholic acid) | 0.241 (0.122; 0.372) | **0.105 (0.065; 0.155)** | **0.075 (0.053; 0.107)** | 0.181 (0.097; 0.264) | 0.096 (0.072; 0.162) | 0.126 (0.097; 0.194) |
| **LCA** (lithocholic acid) | 0.012 (0.007; 0.022) | **0.004 (0.004; 0.008)** | **0.003 (0.002; 0.005)** | 0.01 (0.006; 0.013) | 0.007 (0.004; 0.01) | 0.007 (0.005; 0.013) |
| **Bile acid conjugates** | | | | | | |
| **GCA** (glycocholic acid) | 0.041 (0.03; 0.146) | **0.017 (0.012; 0.035)** | **0.013 (0.009; 0.021)** | 0.033 (0.023; 0.079) | 0.029 (0.017; 0.04) | 0.038 (0.017; 0.1) |
| **GCDCA** (glycochenodeoxycholic acid) | 0.26 (0.122; 0.371) | **0.046 (0.034; 0.08)** | **0.037 (0.029; 0.05)** | 0.182 (0.121; 0.23) | 0.1 (0.061; 0.212) | 0.128 (0.059; 0.304) |
| **GDCA** (glycodeoxycholic acid) | 0.07 (0.043; 0.124) | **0.019 (0.012; 0.034)** | **0.014 (0.007; 0.019)** | 0.053 (0.019; 0.078) | 0.033 (0.027; 0.073) | 0.041 (0.027; 0.073) |
| **GLCA** (glycolithocholic acid) | 0.014 (0.006; 0.025) | **0.005 (0.003; 0.007)** | **0.003 (0.002; 0.004)** | 0.011 (0.006; 0.015) | 0.007 (0.005; 0.018) | 0.007 (0.005; 0.018) |
| **GUDCA** (glycoursodeoxycholic acid) | 0.048 (0.022; 0.085) | **0.013 (0.009; 0.022)** | **0.01 (0.008; 0.015)** | 0.044 (0.018; 0.065) | 0.034 (0,014; 0.053) | 0.038 (0.015; 0.085) |
| **HDCA** (hyodeoxycholic acid) | 0.042 (0.025; 0.077) | 0.029 (0.014; 0.042) | **0.019 (0.011; 0.032)** | 0.034 (0.022; 0.044) | 0.027 (0.020; 0.031) | 0.028 (0.02; 0.034) |
| **TCDCA** (taurochenodeoxycholic acid) | 0.021 (0.013; 0.045) | 0.01 (0.008; 0.013) | **0.007 (0.006; 0.008)** | 0.019 (0.010; 0.035) | 0.013 (0.008; 0.031) | 0.020 (0.013; 0.043) |
| **Oxylipin precursors** | | | | | | |
| **AA** (arachidonic acid) | 3.48 (2.78; 3.94) | 2.87 (2.26; 3.74) | **2.61 (2.01; 3.24)** | 3.32 (2.87; 3.65) | 3.56 (3.09; 4.11) | 3.73 (3.60; 4.23) |
| **EPA** (eicosapentaeonic acid) | 0.59 (0.44; 0.7) | **0.42 (0.3; 0.55)** | **0.37 (0.25; 0.53)** | 0.54 (0.47; 0.72) | 0.7 (0.51; 0.92) | 0.77 (0.56; 0.92) |
| **CYP oxylipins from AA** | | | | | | |
| **5,6-DHET** (5,6-dihydroxy-8,11,14-eicosatrienoic acid) | 0.00025 (0.00018; 0.00033) | 0.0002 (0.00015; 0.00024) | **0.00019 (0.00014; 0.00024)** | 0.00026 (0.0002; 0.00031) | 0.00029 (0.00019; 0.00036) | 0.00032 (0.00026; 0.00039) |
| **11,12-DHET** (11,12-dihydroxy-5,8,14-eicosatrienoic acid) | 0.00045 (0.0004; 0.00054) | **0.00039 (0.00032; 0.00047)** | **0.00035 (0.00029; 0.00041)** | 0.00042 (0.00038; 0.00047) | 0.00054 (0.0004; 0.0006) | 0.00052 (0.00049; 0.00062) |
| **14,15-DHET** (14,15-dihydroxy-5,8,11-eicosatrienoic acid) | 0.00055 (0.00049; 0.00061) | **0.00044 (0.00038; 0.00053)** | **0.00042 (0.00037; 0.0005)** | 0.00048 (0.00047; 0.00054) | 0.00057 (0.00051; 0.00065) | 0.00059 (0.00056; 0.00065) |
| **CYP oxylipins from EPA** | | | | | | |
| **17,18-DiHETE** (17,18-dihydroxy-5,8,11,14-eicosatetraenoic acid) | 0.00505 (0.00431; 0.00646) | 0.00414 (0.00343; 0.00494) | **0.00366 (0.00314; 0.00476)** | 0.00432 (0.0034; 0.00513) | 0.00468 (0.00352; 0.00641) | 0.00527 (0.00425; 0.0069) |
| **CYP oxylipins from LA** | | | | | | |
| **9,10- DiHOME** (9,10-dihydroxy-12-octadecenoic acid) | 0.00232 (0.00156; 0.00327) | 0.00139 (0.00102; 0.00208) | **0.00135 (0.00101; 0.00223)** | 0.00207 (0.00144; 0.00235) | 0.00223 (0.00178; 0.0036) | 0.00233 (0.00186; 0.0031) |
| **12,13-DiHOME** (12,13-dihydroxyoctadec-9-enoic acid) | 0.00591 (0,00384; 0,00767) | 0.0033 (0.00257; 0.00513) | **0.00333 (0.00249; 0.00456)** | 0.00405 (0.0036; 0.00621) | 0.00493 (0.00377; 0.00664) | 0.00518 (0.00328; 0.00646) |
| **9,10-EpOME** (9,10-epoxy-12-octadecenoic acid) | 0,00086 (0,00068; 0,00109) | **0.00055 (0.00039; 0.00082)** | 0.00064 (0.00043; 0.00086) | 0.0009 (0.00067; 0.00128) | 0.00088 (0.00077; 0.00137) | 0.00088 (0.00076; 0.00115) |
| **12,13-EpOME** (12,13-epoxy-9-octadecenoic acid) | 0.00241 (0.00186; 0.00363) | **0.00135 (0.00103; 0.00236)** | 0.00182 (0.00142; 0.00258) | 0.00242 (0.00169; 0.00355) | 0.00228 (0.00179; 0.00322) | 0.00244 (0.0018; 0.00283) |
| **LOX oxylipin from ɑ-LA** | | | | | | |
| **9-HOTrE** (9-hydroxy-10,12,15-octadecatrienoic acid) | 0.00048 (0.00037; 0.00073) | **0.00029 (0.00015; 0.0004)** | **0.00029 (0.00022; 0.00041)** | 0.00044 (0.00036; 0.00066) | 0,00059 (0.00047; 0.00074) | 0.00058 (0.00043; 0.00065) |
| **LOX oxylipin from AA** | | | | | | |
| **5-HETE** (5-hydroxy-6,8,11,14-eicosatetraenoic acid) | 0.00047 (0.00033; 0.00072) | **0.00035 (0.00027; 0.00051)** | **0.00033 (0.00024; 0.00056)** | 0.00053 (0.00042; 0.00061) | 0.0007 (0.00049; 0.00081) | 0.00068 (0.00047; 0.00082) |
| **LOX oxylipin from LA** | | | | | | |
| **9-HODE** (9-hydroxy-10,12-octadecadienoic acid) | 0.00421 (0.00292; 0.00681) | **0.00261 (0.00192; 0.00442)** | **0.00269 (0.00169; 0.00404)** | 0.00432 (0.00287; 0.00658) | 0.00477 (0.00399; 0.00646) | 0.00487 (0.00413; 0.0068) |
| **13-KODE** (13-Oxo-9,11-octadecadienoic acid) | 0.00198 (0.00148; 0.00242) | 0.00148 (0.00104; 0.0019) | **0.00132 (0.0011; 0.00182)** | 0.0017 (0.00144; 0.00189) | 0.0019 (0.00162; 0.00221) | 0.00185 (0.00159; 0.0023) |

Reported values are in µmol l-1, median [interquartile range]. Statistically significant change from baseline in comparison to placebo is bolded. Bile acid precursor, bile acids and conjugates rounded at 3 decimal places, oxylipin precursors at 2, oxylipins at 5. AA, arachidonic acid; EPA, eicosapentaenoic acid; LA, linoleic acid.

**Appendix B, supplementary Table 4.** Absolute analyte concentrations, propofol, sevoflurane, and S-ketamine *vs*. placebo.

|  | **Propofol** | | | **Placebo** | | |
| --- | --- | --- | --- | --- | --- | --- |
| **Oxylipin precursors** | **Timepoint 1** | **Timepoint 2** | **Timepoint 3** | **Timepoint 1** | **Timepoint 2** | **Timepoint 3** |
| **EPA** (eicosapentaeonic acid) | 0.58 (0.43; 0.71) | **0.49 (0.33; 0.56)** | 0.62 (0.44; 0.78) | 0.54 (0.47; 0.72) | 0.7 (0.51; 0.92) | 0.77 (0.56; 0.92) |
| **CYP oxylipins from LA** | | | | | | |
| **9,10- DiHOME** (9,10-dihydroxy-12-octadecenoic acid) | 0.00225 (0.00161; 0.00345) | **0.01714 (0.00952; 0.01937)** | 0.0041 (0.00307; 0.00583) | 0.00207 (0.00144; 0.00235) | 0.00223 (0.00178; 0.0036) | 0.00233 (0.00186; 0.0031) |
| **12,13-DiHOME** (12,13-dihydroxyoctadec-9-enoic acid) | 0.00492 (0.00362; 0.00861) | **0.02235 (0.01305; 0.02608)** | 0.00722 (0.00549; 0.00947) | 0.00405 (0.0036; 0.00621) | 0.00493 (0.00377; 0.00664) | 0.00518 (0.00328; 0.00646) |
| **LOX oxylipin from ɑ-LA** | | | | | | |
| **9-HOTrE** (9-hydroxy-10,12,15-octadecatrienoic acid) | 0.00053 (0.00042; 0.00082) | **0.0019 (0.00158; 0.00244)** | 0.00066 (0.00053; 0.00088) | 0.00044 (0.00036; 0.00066) | 0.00059 (0.00047; 0.00074) | 0.00058 (0.00043; 0.00065) |
| **LOX oxylipin from LA** | | | | | | |
| **9-HODE** (9-hydroxy-10,12-octadecadienoic acid) | 0.005 (0.00298; 0.00724) | **0.01089 (0.00928; 0.01294)** | 0.00521 (0.00455; 0.00687) | 0.00432 (0.00287; 0.00658) | 0.00477 (0.00399; 0.00646) | 0.00487 (0.00413; 0.0068) |
|  |  |  |  |  |  |  |
|  | **Sevoflurane** | | | **Placebo** | | |
| **Bile acid conjugates** | **Timepoint 1** | **Timepoint 2** | **Timepoint 3** | **Timepoint 1** | **Timepoint 2** | **Timepoint 3** |
| **TUDCA** (tauroursodeoxycholic acid) | 0.008 (0.008; 0.012) | **0.007 (0.005; 0.008)** | 0.007 (0.006; 0.011) | 0.007 (0.006; 0.008) | 0.007 (0.007; 0.012) | 0.009 (0.007; 0.012) |
| **CYP oxylipins from EPA** | | | | | | |
| **17,18-DiHETE** (17,18-dihydroxy-5,8,11,14-eicosatetraenoic acid) | 0.00492 (0.00362; 0.00674) | **0.00778 (0.00646; 0.01019)** | 0.00529 (0.00405; 0.00732) | 0.00432 (0.0034; 0.00513) | 0.00468 (0.00352; 0.00641) | 0.00527 (0.00425; 0.0069) |
|  |  |  |  |  |  |  |
|  | **S-ketamine** | | | **Placebo** | | |
| **Precursor for primary bile acid synthesis** | **Timepoint 1** | **Timepoint 2** | **Timepoint 3** | **Timepoint 1** | **Timepoint 2** | **Timepoint 3** |
| **HCO** (7α-Hydroxy-4-cholesten-3-one) | 0.047 (0.031; 0.057) | 0.032 (0.028; 0.044) | **0.025 (0.019; 0.039)** | 0.033 (0.018; 0.051) | 0.028 (0.016; 0.037) | 0.032 (0.022; 0.048) |

Reported values are in µmol l-1, median [interquartile range]. Statistically significant change from baseline in comparison to placebo is bolded. Bile acid precursor, bile acids and conjugates rounded at 3 decimal places, oxylipin precursors at 2, oxylipins at 5. EPA, eicosapentaenoic acid; LA, linoleic acid.

**Appendix B, supplementary Figure 1-4., Forest plots of anaesthetic comparisons**

Dexmedetomidine, propofol, sevoflurane, and S-Ketamine *vs.* placebo, and all anaesthetic-anaesthetic comparisons of all analysed metabolites. Change is reported in SDS with 95% confidence intervals. For all anaesthetic-anaesthetic comparisons. The vertical lines depict 0 and +/- 1 SD thresholds. The colour coding represents the changes in timepoints 1 *vs.* 2 and 1 *vs.* 3, the significant changes after Bonferroni correction are highlighted. HCO, 7α-Hydroxy-4-cholesten-3-one; CA, cholic acid; CDCA, chenodeoxycholic acid; DCA, deoxycholic acid; LCA, lithocholic acid acid; UDCA, ursodesoxycholic acid; GCA, glycocholic acid; GCDCA, glycochenodeoxycholic acid; GDCA, glycodeoxycholic acid; GLCA, glycolithocholic acid; GUDCA, glycoursodeoxycholic acid; HDCA, hyodeoxycholic acid; TCA, taurocholic acid; TCDCA, taurochenodeoxycholic acid acid; TDCA, taurodeoxycholic acid; TUDCA, tauroursodeoxycholic acid; AA, arachidonic acid; EPA, eicosapentaeonic acid; 12-HHT, 12-hydroxy-5,8,10-heptadecatrienoic acid; PGD2, prostaglandin D2; PGE2, prostaglandin E2; TXB2, thromboxane B2; 5,6-DHET, 5,6-dihydroxy-8,11,14-eicosatrienoic acid; 8,9-DHET, 8,9-dihydroxy-5,11,14-eicosatrienoic acid; 11,12-DHET, 11,12-dihydroxy-5,8,14-eicosatrienoic acid; 14,15-DHET, 14,15-dihydroxy-5,8,11-eicosatrienoic acid; 16-HETE, 16-hydroxy-5,8,11,14-eicosatetraenoic acid; 18-HETE, 18-hydroxy-5,8,11,14-eicosatetraenoic acid; 20-COOH-AA, 5,8,11,14-eicosatetraenedioic acid; 5,6-DiHETE, 5,6-dihydroxy-8,11,14,17-eicosatetraenoic acid; 17,18-DiHETE, 17,18-dihydroxy-5,8,11,14-eicosatetraenoic acid; 9,10-DiHOME, 9,10-dihydroxy-12-octadecenoic acid; 12,13-DiHOME, 12,13-dihydroxyoctadec-9-enoic acid; 9,10-EpOME, 9,10-epoxy-12-octadecenoic acid; 12,13-EpOME, 12,13-epoxy-9-octadecenoic acid; 9-HOTrE, 9-hydroxy-10,12,15-octadecatrienoic acid; 5-HETE, 5-hydroxy-6,8,11,14-eicosatetraenoic acid; 11-HETE, 11-hydroxy-5,8,12,14-eicosatetraenoic acid; 12-HETE, 12-hydroxy-5,8,10,14-eicosatetraenoic acid; 15-HETE, 15-hydroxy-5,8,11,13-eicosatetraenoic acid; 14-HDoHE, 14-hydroxy docosahexaenoic acid; 12-HEPE, 12-hydroxy-5,8,10,14,17-eicosapentaenoic acid; 9-HpODE, 9-hydroperoxyoctadeca-10,12-dienoic acid; 9-HODE, 9-hydroxy-10,12-octadecadienoic acid; 13-KODE, 13-keto-9,11,-octadecadienoic acid; LA, linoleic acid; alpha-LA, alpha-linoleic acid; DHA, docosahexaenoic acid.
